# Two Cases of Compound Depressed Fracture of Frontal Bone

**Published:** 1897-03

**Authors:** Arthur W. Prichard

**Affiliations:** Surgeon to the Bristol Royal Infirmary


### TWO CASES OF COMPOUND DEPRESSED FRACTURE OF FRONTAL BONE.

BY

ARTHUR W. PRICHARD, M.R.C.S. Eng.,
*Surgeon to the Bristol Royal Infirmary.*

---

I THINK that the two cases I am about to briefly narrate will be found interesting, the most important point about them being the somewhat unexpected recovery of the patients,—one after going through the risks of a bad accident, two trephinings and a tedious hernia cerebri, and the second after having had the lower half of his frontal bone kicked in.

CASE 1.—W. H., a boy 13 years old. On December 12th, 1895, about 6 p.m., as he was stepping off the pavement, he received on his forehead a blow from the shaft of a baker's cart which knocked him down. He remembers being driven to the Infirmary and being carried to the ward, and was able to give his name and address; and he recognised his father about an hour later, but afterwards he lapsed into semi-consciousness, and was in that state when I saw him between 9 and 10. He was then restless, and had vomited; there were no twitchings or convulsions; pupils were equal and reacted to light; there was no bleeding from nose or ears. I found a depressed fracture of the frontal bone, with a wound near the middle line just above the nose, the wound being vertical in direction and 1½ inches long. The patient was put under chloroform, and I made an incision horizontally for about two inches on each side from the lowest part of the wound, and turning back the flaps, I found a stellate fracture radiating from the glabella and extending above the left orbital plate further than on the right side, the most central part being most depressed. I trephined with a medium-sized trephine just to the left of the middle line and elevated satisfactorily the whole of the depressed bone. The crista galli and the superior longitudinal sinus prevented my passing the elevator right across the middle, but the fragments on the right were movable, and when I left the patient I thought all was tolerably level. The dura mater apparently was not broken.

Next day patient was comfortable and conscious. He vomited once, but there were no signs of intra-cranial mischief; the pins which I had used to check hemorrhage and bring together the wound, and the drainage tube, were removed.

During the ensuing week headache, high temperature, and delirium with great irritability came on, and on the 20th, one week from the time of the accident, he had a distinct fit which lasted twelve minutes; the twitching began in the left arm and left leg and the face worked all over. His left eye squinted upwards and inwards. He had another fit in the night, the left side again being chiefly affected.

Next day I trephined him on the right of the middle line just above the orbital plate. The dura mater bulged and at one part appeared almost yellow. I passed a hypodermic needle in, and as I only withdrew a few drops of serous fluid, I made a crucial incision through the dura mater. No pus could be found, and I stitched up the wound in the dura mater and left drainage tubes in the external wounds.

He improved immediately after the operation, and a week later all sutures were removed, and patient was so far recovered that he was found to be sitting up in his bed beating a toy drum—a proof that there were no signs of meningitis. The wound on the right side, however, did not heal soundly, granulations sprang up in it and pus in considerable quantity manifested itself. A hernia cerebri now gradually developed in the wound, commencing about the ninth day after the second operation, and it increased in spite of all we did to suppress it. A pad was made of a watch-glass filled with plaster-of-Paris, and kept on by an elastic bandage outside the lint and under the wool dressings that covered the wound; iodoform, boric acid, and other antiseptics were tried, and the hernia was once shaved off. This consisted microscopically of a meshwork of processes of cells containing small cells—neuroglia chiefly and granulation tissue—but there were no distinct nervous elements found.

The treatment was unsatisfactory; the hernia grew again, and the patient showed signs of considerable irritability, although his bodily health somewhat improved.

A month after the second operation I resolved to try and cure the hernia by operation. I made a clean oval cut round the hernia through the much-thickened skin of the forehead, and removed the granulation tissue all round the growth. Then I shaved off the hernia and brought together the longer sides of the wound by harelip pins, putting in a very small drain. This plan succeeded very well, and the wound was firmly healed in ten days, but the area where the hernia had been remained soft for some time. I kept a vulcanite plate for almost six months on it for protection, and the boy was able to go to school; and for the last few months he has been without any apparatus, and is able to help his father in his work.

Case 2.—A man, aged 22, by occupation a butcher, but who occasionally acted as stableman was seen to be taking home a horse after a performance at Carré's circus, and was seen to unmercifully beat it. Nothing more was seen till he was found lying at the heels of the horse in the stable with his head much injured. He was roused and with help walked to the Infirmary, and was admitted about 11 p.m. He was conscious and stated that a man whom he did not know threw a brick at him, which struck him on the forehead and he fell under the horse.

When I saw him very shortly afterwards he was lying on the operation table, with the lower half of the frontal bone completely driven in. There was a much-contused wound just above the bridge of his nose, and another communicating with it across the forehead about the size and shape of the front part of the shoe of a horse. There was brain-matter protruding from both sides of the wound, so that both hemispheres were lacerated, the superior longitudinal sinus was torn, and considerable bleeding resulted. The patient vomited much blood, and blood came from his right ear and freely from his nose. The disfigurement was further increased by a huge swelling at the angle of the jaw, but this I subsequently learnt was of old standing. So terrible did his injuries seem that I doubted for a minute whether to touch him or leave him to die, but I chose the former alternative. I made an incision nearly from one external angular process to the other and a medium one up the forehead, and having stopped external bleeding, trephined the left side of the frontal bone just above the fracture, and I sawed off a long point of the squamous portion of the right temporal bone that was presenting and which could not be replaced. By these means I had room enough for the elevator to raise all the depressed bone; with my finger I removed brain-matter that was crushed and protruding from the ruptured dura mater on either side. The superior longitudinal sinus bled freely. Having elevated the bone and having pushed back as well as I could the squamous portion of the right temporal bone, I brought the skin together by numerous fine harelip pins and put in several drainage tubes, and packed the forehead in absorbent wool and sent the patient back to bed.

Next day he was perfectly conscious, but had very great pain in the head, which was relieved by morphia. There was discharge of blood-stained cerebro-spinal fluid from the right ear, the vomit was tinged with blood, and there was copious discharge from the nose. He remained in this state for several days, drinking milk readily; and cerebro-spinal fluid came freely from his nose and ear. The only cerebral symptom was slight delirium in the evening after there had been a necessary visit from the police to enquire into the charge the patient had made against his assailant.

On the fourth day when taking out some of the pins I occasioned free arterial hemorrhage, and I had to stop it by inserting another pin under the vessel.

A week later, eleven days from the day of the operation, the wounds were nearly healed. A small amount of pus came from the granulations at the lower end of the middle wound, where the greatest crushing of the soft parts was.

The patient got up on the twentieth day; and went out four weeks after the accident, against advice. All wounds had healed, and he said he felt quite well and had no headaches. He seemed perfectly rational. There are soft and pulsating areas on his forehead, on which he has been cautioned that a blow might be very serious.

Such is the history of my two patients, and I bring them forward because I believe in the advantage of mentioning details of cases. I have very little comment to make. At one period in each case I was almost hopeless that the patient would recover.

In the first case the bad prognosis with which all cases of hernia cerebri are connected, and the apparently futile attempts which I made to cure it by means of pressure and antiseptics, gave me very little hope of the patient's life. A plastic operation, such as succeeded in this boy, is what I shall recommend in the future. It is an interesting point to make out why hernia cerebri occurred in one and not in the other of my two cases. Was it that in the case of the boy the wound that I made in the dura mater at the second operation became septic from an unhealthy condition of the first wound? or was it that in his case the wound in the dura mater was small as compared with the larger lacerations in the other? I am inclined to think that the aseptic course run by the healing of the wounds in the man's head had most to do with the non-appearance of a hernia cerebri.

The severity of the injury in the second case, the large portion of bone that was driven in, with crushed brain-matter escaping from at least two places, the long point of the squamous portion of the temporal that was protruding,—these injuries, in addition to the sure signs of extensive fracture of the base, made me doubt whether it was of any use trying any operation at all. I may be criticised for my opinion, but I think his recovery was in a great measure due to the very free bleeding that occurred before and at the time of operation, and which continued through the drainage tubes into the dressings afterwards.

